# Expression and protein localisation of *IGF2 *in the marsupial placenta

**DOI:** 10.1186/1471-213X-8-17

**Published:** 2008-02-20

**Authors:** Eleanor I Ager, Andrew J Pask, Geoff Shaw, Marilyn B Renfree

**Affiliations:** 1 Department of Zoology, The University of Melbourne, Melbourne, Victoria, 3010, Australia

## Abstract

**Background:**

In eutherian mammals, genomic imprinting is critical for normal placentation and embryo survival. *Insulin-like growth factor 2 *(*IGF2*) is imprinted in the placenta of both eutherians and marsupials, but its function, or that of any imprinted gene, has not been investigated in any marsupial. This study examines the role of *IGF2 *in the yolk sac placenta of the tammar wallaby, *Macropus eugenii*.

**Results:**

*IGF2 *mRNA and protein were produced in the marsupial placenta. Both IGF2 receptors were present in the placenta, and presumably mediate IGF2 mitogenic actions. *IGF2 *mRNA levels were highest in the vascular region of the yolk sac placenta. IGF2 increased *vascular endothelial growth factor *expression in placental explant cultures, suggesting that IGF2 promotes vascularisation of the yolk sac.

**Conclusion:**

This is the first demonstration of a physiological role for any imprinted gene in marsupial placentation. The conserved imprinting of *IGF2* in this marsupial and in all eutherian species so far investigated, but not in monotremes, suggests that imprinting of this gene may have originated in the placenta of the therian ancestor.

## Background

Eutherians and marsupials (therian mammals) diverged between 125 and 145 million years ago [1,2] and both develop a placenta to support embryonic growth and development. Mammalian placental structures arise from the union of either yolk sac or allantois with the chorion but many mammals possess both kinds placentation [3-6]. The majority of marsupials, however, rely exclusively on a chorio-vitelline or yolk sac placenta which consists of two structurally distinct regions. The avascular, bilaminar yolk sac (BYS) is presumed to be the primary site of nutrient exchange between mother and young while the vascular, trilaminar yolk sac (TYS) acts as the primary route for gas exchange [7-10].

Genomic imprinting, an epigenetic phenomenon in which a single allele of a gene is active from only one parental chromosome has, amongst mammals, so far only been found in therians [11-15]. Almost all imprinted genes identified affect growth or are embryonic lethal when mutated. The parental conflict hypothesis is the most widely accepted of many hypotheses explaining imprinting and suggests that it evolved as a consequence of divergent selection on parental genes controlling maternal nutrient transfer *in utero *[16,17]. Since the placenta mediates the transfer of nutrients between mother and young, it is an important site for the expression of imprinted genes. Indeed, several hypotheses suggest that placentation and genomic imprinting may have co-evolved [18-21].

Although many imprinted genes fit the predictions of the conflict hypothesis, its applicability to marsupials and, therefore, its broader relevance has not been investigated. *Insulin-like growth factor 2 *(*Igf2*) gene is paternally expressed in the mouse [22,23] and in at least two marsupials, the South American grey short-tailed opossum (*Monodelphis domestica*) [11] and the Australian tammar wallaby (*Macropus eugenii*), in which it is paternally expressed in both the fetus and placenta [14]. Another three eutherian imprinted genes (*IGF2R*,*PEG1/MEST *and *PEG10*) are also imprinted in the tammar [14,15] and in the North American opossum, *Didelphis virginiana *[12]. However, the expression, protein localisation, and function of *IGF2*, or any imprinted gene, have not been described in the marsupial yolk sac placenta.

IGF2 promotes cellular hypertrophy, cell survival, and hyperplasia [24,25] and is highly conserved in vertebrates [26,27]. In mammals, the availability and action of IGF2 is mediated by a family of six binding proteins (IGF-BPs) and three receptors (IGF2R, IGF1R, and IR), many of which are expressed in placental and uterine tissues [28-32]. Most of the metabolic and mitogenic effects of IGF2 are mediated through the IGF1R [33]. The primary role of IGF2R during eutherian development is to limit the availability of IGF2 by its internalisation and lysosomal degradation [34,35].

IGF2 can have endocrine, paracrine, or autocrine actions, with the latter two particularly important for fetal development [36-38]. *Igf2*-knockout mice demonstrate its necessity for chorioallantoic placentation [39-43]. IGF2 has been implicated in several aspects of placental development, including blood vessel formation [31], trophoblast invasion [29,32,44], nutrient transfer [39,41,42], and differentiation [39,40]. IGF2 also contributes to the transcriptional regulation of several genes including *VEGF *(*vascular endothelial growth factor*) and this interaction may be important for placental development [45-47]. IGF2 mutations are associated with gestational diseases such as pre-eclampsia in which angiogenesis is disrupted [48], possibly as a result of increased expression of *VEGF*.

If *IGF2 *imprinting evolved as a consequence of its functional importance in therian placentation then it should, in addition to being imprinted in marsupials, also function in the marsupial placenta. The present study describes the temporal expression of *IGF2 *mRNA and the location of IGF2 and two of its receptor proteins (IGF1R and IGF2R) in the yolk sac placenta of the tammar wallaby. To investigate the functional importance of IGF2 in the placenta, yolk sac explants were cultured *in vitro *in the presence or absence of exogenous IGF2 and its effects on *VEGF *expression in the yolk sac examined.

## Results

### Protein localisation in the yolk sac

**Figure 1:**
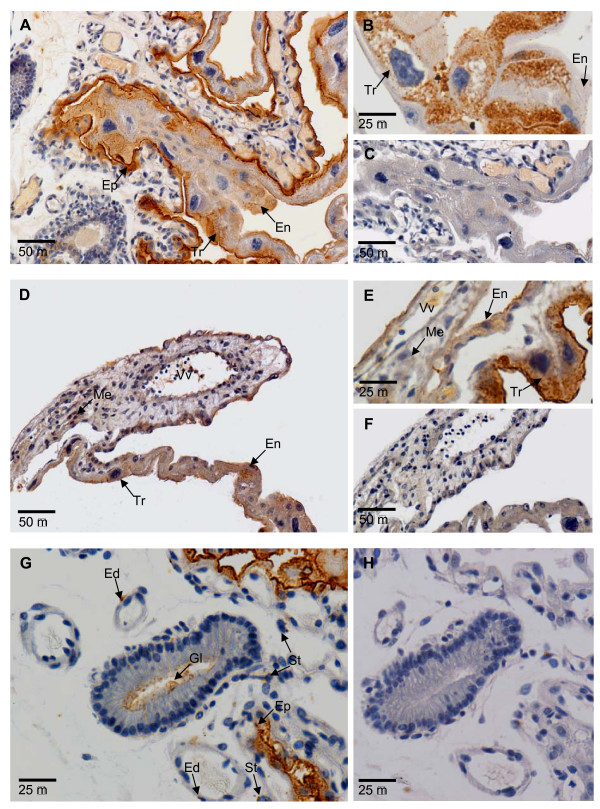
IGF2 protein in the bilaminar (A and B) and trilaminar (D and E) yolk sac at day 25–26 of gestation. IgG negative controls for the bilaminar (C) and trilaminar (F) yolk sac. Staining was strongest in the trophoblast (Tr), but some endodermal (En) cells of the yolk sac placenta also stained. Staining was generally stronger in the trilaminar yolk sac and in both portions of the yolk sac staining increased later in gestation (see Fig. 2). Strong staining can be seen in the uterine epithelium (Ep) immediately adjacent to the bilaminar (avascular) yolk sac placenta. There was little staining in the mesenchyme (Me) and endothelium of large vitelline vessels (Vv) of the trilaminar (vascular) yolk sac placenta. Some stromal (St) and endothelial cells (Ed) in the maternal endometrium also stained (G and IgG negative H), as did the uterine epithelium (Ep) and some endometrial glands (Gl). Scale bar is shown at the bottom left of each image.

IGF2 was largely cytoplasmic in all tissues tested. In the uterus, IGF2 protein was localised in the cytoplasm of glandular cells in the endometrium and in the uterine epithelium. Accumulated staining was observed in the lumen of some, but not all, uterine glands and in a few cells of the uterine stroma (Fig. 1). In the yolk sac, IGF2 protein was detected in the trophoblast and yolk sac endoderm of bilaminar and trilaminar regions, but rarely in the mesenchymal or endothelial cells of vitelline vessels (Fig. 1).

**Figure 2:**
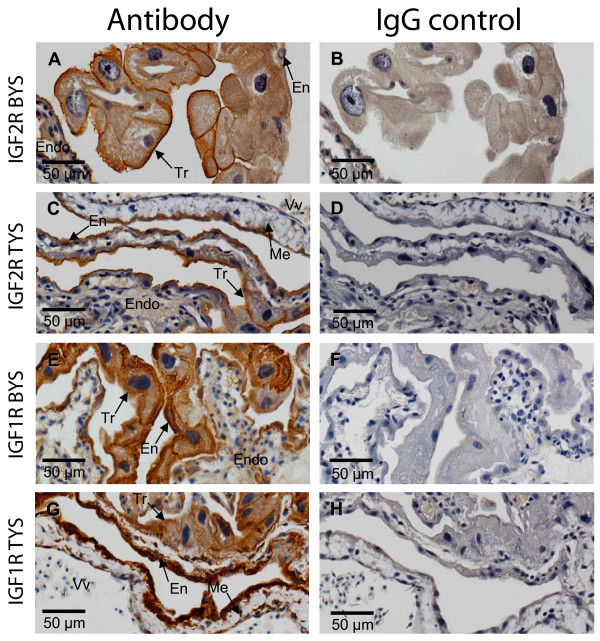
IGF2R (A & C) and IGF1R (E & G) protein in the bilaminar (BYS; A & E) and trilaminar (TYS; C & G) yolk sac at day 25. Appropriate IgG antibody negative controls for IGF2R and IGF1R antibodies are shown (B, D, F, & H). IGF2R staining was strongest in the trophoblast (Tr), with lighter staining in the yolk sac endoderm (En) and little or no staining in the mesenchyme (Me) surrounding vitelline vessels (Vv). IGF2R staining was localised in the cytoplasm and cell membrane. IGF1R stained all yolk sac cell types. Both antibodies also stained the uterine epithelium and some stromal cells in the endometrium (Endo). Scale bar is shown at the bottom left of each image.

**Figure 3:**
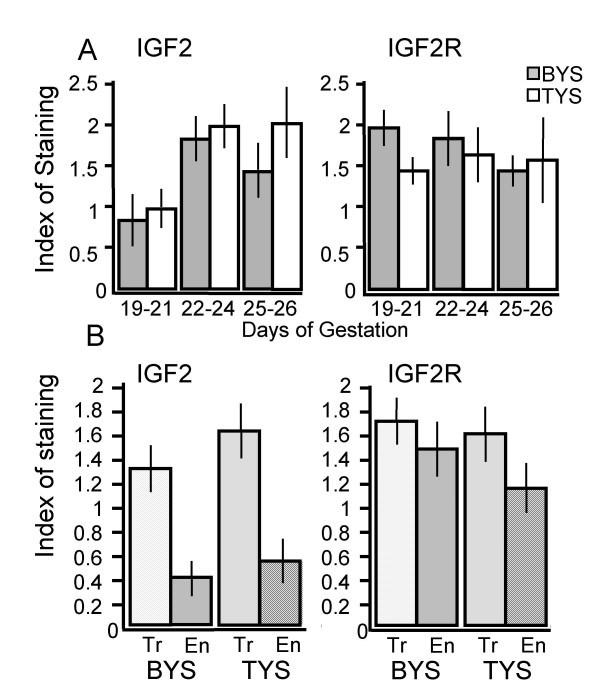
Intensity of staining to IGF2 and IGF2R antibodies in the yolk sac trophoblast during the final third of gestation (A). The intensity of staining was measured subjectively as described in the experimental procedures. The bilaminar (BYS) (shaded bars) and trilaminar yolk sac (TYS) (open bars) of matched samples were assessed independently. Samples were grouped into days 19–21 (n = 4), 22–24 (n = 5), and 25–26 (n = 4). Staining intensity to the IGF2 antibody was consistently stronger in the TYS, especially at days 25–26. Staining by the IGF2 antibody was notably lighter at days 19–21 than later stages (days 22 to 26). Staining by the IGF2R antibody did not differ notably between the bilaminar and trilaminar yolk sac, nor were there marked differences corresponding to developmental stage. Intensity of staining by IGF2 and IGF2R antibodies in yolk sac cells (B). Staining intensity was noticeably higher in the trophoblast (Tr) (stippled bars) than in the yolk sac endoderm (En) (striped bars) of the bilaminar (BYS) and trilaminar (TYS) for IGF2, but not IGF2R. The staining intensity represents the average for fetal stages between days 19 and 26 (n = 13). Light background staining with the IgG antibody negative control in the yolk sac endoderm was taken into account when judging the staining intensity of the yolk sac endoderm for IGF2 and IGF2R antibodies.

IGF2R protein co-localised with IGF2 in the yolk sac, but was also detected in the cell membrane in addition to the cytoplasm (Fig. 2). Unlike IGF2, IGF2R staining was similar in both the bilaminar and trilaminar yolk sac and it did not markedly change over the developmental period examined (Fig. 3A). Staining for IGF2R was more restricted than IGF2 in the endometrium, with immuno-reactivity limited to the uterine epithelium. All cells in the bilaminar and trilaminar yolk sac, including the mesenchyme and endothelium, reacted with the IGF1R antibody (Fig. 2). There was no staining in the IgG antibody or no-antibody negative controls. Staining for IGF1R was common in the endometrium, with reactivity to the endometrial stroma, endometrial glands, and many endothelial cells.

IGF2 antibody immunoreactivity was stronger in the bilaminar than in the trilaminar yolk sac at all stages examined, but this difference was most notable in the two days before parturition (Fig. 3A). Both the bilaminar and trilaminar yolk sac had less IGF2 immuno-staining between days 19 to 21 than at later stages of pregnancy. IGF2 immunostaining in the trophoblast of the bilaminar and trilaminar yolk sac was consistently stronger than in the yolk sac endoderm (Fig. 3B). Additionally, there was light background staining in the yolk sac endoderm, but not the trophoblast, of IgG antibody negative controls (Fig. 1). However, background staining was not as intense as staining to the IGF2 antibody. Although there was stronger staining of IGF2R in the trophoblast, the intensity of staining was not markedly different from the yolk sac endoderm (Fig. 3B).

### Confirming antibody specificity

**Figure 4:**
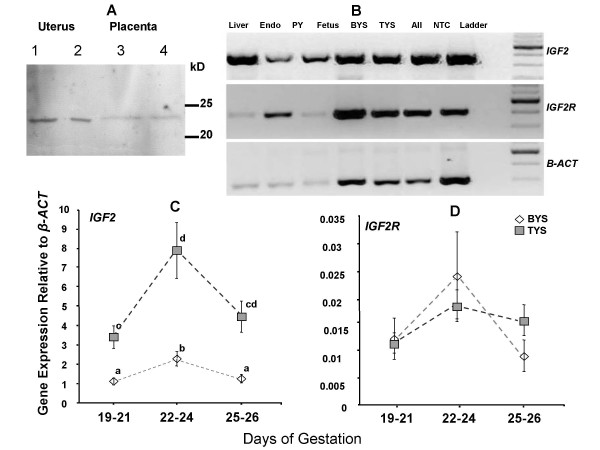
(A) IGF2 Western blot. A single band was detected at approximately 23 kD, consistent with predicted the protein size. Non-quantitative (B) and quantitative (C) *IGF2 *and *IGF2R *RT-PCR. Tissues include adult liver, endometrium (endo), pouch young tail (PY), fetal body (fetus), bilaminar yolk sac (BYS,), trilaminar yolk sac (TYS) and allantois (all), a "no template" control (NTC) is also shown. Only the BYS and TYS were examined quantitatively and stages examined were grouped; 19–21 (n = 8), 22–24 (n = 7), and 25–26 (n = 6). *IGF2 *mRNA was expressed on both TYS (stippled squares), and BYS (open diamonds) but was higher in the TYS at all stages. *IGF2 *expression increased between days 19–21 and 22–24. *IGF2R *mRNA levels fluctuated, but the BYS and TYS were not significantly different. Significant differences are shown by superscript letters. Means sharing the same letters are not significantly different (P > 0.05). Means with different superscript letters are significantly different (P ≤ 0.05).

Western blots using protein extracts from both the uterus and placenta detected a single band of approximately 23 kD consistent with predicted protein size for IGF2. This confirmed that the antibody was specific for tammar IGF2, validating the immunohistochemistry (Fig 4A).

### Non-quantitative gene expression

RT-PCR amplified products of the expected size, 422 bp (*IGF2*) and 443 bp (*IGF2R*), which were sequenced to confirm gene identity. BLAST-N on the sequence of these PCR fragments showed high homology with sequences in other species: tammar *IGF2 *showed 95% nucleotide identity to North American opossum *IGF2 *(AY55235.1) and tammar *IGF2R *showed 99% nucleotide identity to the red-necked wallaby, *Macropus rufogriseus IGF2R *(AF339159). *IGF2 *and *IGF2R *mRNA was detected in the bilaminar and trilaminar yolk sac of all stages between day 19 and day 26. Additionally, both genes were expressed in the allantois, adult liver, endometrium, and in the pouch young tail (Fig. 4B).

### Quantitative levels of gene expression in vivo and in vitro

Contamination by primer dimers was eliminated from analysis by reading sample fluorescence above the primer dimer melting temperatures, as indicated by melting curve analyses, thus ensuring C_T _values reflected amplification of the target only. Melting curve analysis and agarose gel electrophoresis also confirmed a single product was obtained for each reaction. β-Actin (*β-ACT) *(the endogenous gene control) had no primer dimers and plates could be read at temperatures required for target genes.

All standard curves were linear over three orders of magnitude of yolk sac cDNA dilutions, indicating that the primers work over a range of cDNA concentrations. A correlation co-efficient above 0.98 was recorded for the standard curve of all genes examined. Standard deviations of C_T _values from triplicate reactions were on average 0.28 of a cycle for *IGF2*, 0.58 for *IGF2R*, and 0.56 for *VEGF*. Therefore, within each triplicate all C_T _values were within 1 cycle of each other. If standard deviation within the triplicate was greater than 1.5, indicating a substantial variation in the estimated C_T_, all data for those individuals were removed from further analyses.

*IGF2 *expression was significantly lower in the bilaminar compared to the trilaminar yolk sac at all stages from days 19 to 26 (Bonferroni adjusted paired t-test, n ≤ 5, α ≤ 0.013) (Fig. 4B). In both regions of the yolk sac there was a significant increase in *IGF2 *expression between days 19 to 21 and days 22 to 24 (Bonferroni adjusted unpaired t-test, n = 7, α 0.009). Further, *IGF2 *declined at term and this was significant in the bilaminar yolk sac (Bonferroni adjusted unpaired t-test, n = 6, α ≤ 0.036). Unlike *IGF2*,*IGF2R *expression was similar in the bilaminar and trilaminar yolk sac and did not change markedly over the gestational period examined (Fig. 4C).

**Figure 5:**
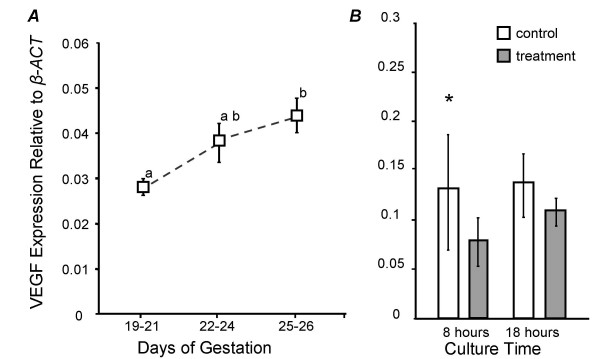
*VEGF *mRNA (open squares) relative to β-actin levels in trilaminar yolk sac during the final third of gestation (A) or *in vitro *(B). *VEGF *expression in the trilaminar yolk sac was examined at stages 19–21 (n = 8), 22–24 (n = 7), and 25–26 (n = 6). A gradual increase in *VEGF *expression is evident and by term (days 25–26) expression was significantly higher than days 19–21 (t-test, one-way, equal variance, P= 0.002, F-test = 0.165). Trilaminar yolk sac explants were cultured for 8 (n = 4) and 18 (n = 5) hours with hr-IGF2 (treatment: stippled bars) or in media only (control: open bars). *VEGF *expression was consistently higher in IGF2 treated explants (see text).

*VEGF *was expressed in the trilaminar yolk sac at all gestational stages examined. A gradual increase in *VEGF *expression was observed for days 19 to 21 through to days 25 to 26 (Fig. 5A). The presence of additional IGF2 (in the form of human-recombinant IGF2 at 100 ng/ml) significantly increased *VEGF *expression in trilaminar yolk sac explants cultured for 8 hours compared to control cultures (one-tailed, one-sample t-test, n = 4, α = 0.023) (Fig. 5B). At 18 hours a similar trend was observed, but was not significant (one-tailed, one-sample t-test, n = 5, α = 0.110). Combining this data with that from the 8 hour treatments gives an overall significant increase in *VEGF *expression when IGF2 was added to the culture medium (ANOVA, n = 9, α = 0.012).

## Discussion

*IGF2 *was expressed in the embryonic, extra-embryonic, and maternal reproductive tissues during the final third of gestation in the tammar. Both mRNA and protein were present in bilaminar and trilaminar regions of the yolk sac, increasing at days 22–24 of the 26.5 day gestation. At all stages expression was higher in the vascular region of the placenta, although there was little IGF2 protein in the yolk sac mesenchyme. IGF2R and IGF1R proteins were in all cells of the placenta, but staining for IGF2R, like IGF2, was minimal in the mesenchyme. The conserved expression of *IGF2 *(and its receptors) in the therian yolk sac suggests that placental expression of *IGF2 *predated or evolved with its imprinting in this tissue. *VEGF *was also expressed in the yolk sac placenta of the tammar. *VEGF *expression increased significantly after addition of IGF2 to cultures of trilaminar yolk sac explants, suggesting that the function of IGF2 in stimulating angiogenesis may be a conserved feature of mammalian placentation.

### Expression and function of IGF2 in the bilaminar and trilaminar yolk sac

In many eutherians, *IGF2 *mRNA is abundant in trophoblast-derived cell lineages, yolk-sac endoderm and mesoderm, and chorioallantoic mesoderm of eutherians [31,32]. Similarly, tammar IGF2 protein was abundant in analogous cell lineages of the yolk sac – the trophoblast and yolk sac endoderm. However, while *IGF2 *mRNA expression is high in many mesodermal tissues in eutherians, there was little IGF2 protein detected in the yolk sac mesenchyme of the tammar. However, *IGF2 *mRNA expression in the trilaminar yolk sac was higher than in the bilaminar yolk sac

IGF2 can act as both a mitogen and a differentiation factor, which it does by triggering different signalling pathways [49,50]. The expression of *IGF2 *mRNA and protein, as well as the co-localisation of both IGF receptors in the tammar yolk sac, provides evidence of its function in this tissue. Basal *IGF2 *expression in the bilaminar yolk sac suggests a constitutive mitogenic role that is likely shared with the trilaminar yolk sac, and is consistent with the localisation of IGF1R, the primary mediator of IGF2 mitogenic activity throughout the yolk sac. High *IGF2 *expression in the trilaminar yolk sac may reflect high rates of proliferation in this region during late gestation [7,9,51,52]. Between days 13 and 26 the trilaminar yolk sac rapidly expands, from approximately 1/20 of the yolk sac surface to 1/2 by the end of gestation [9,52,53].

Igf2 induction of the mesoderm can be independent of Igf2 regulation of cellular proliferation [54]. The abundance of IGF2 binding proteins in yolk sac blood vessels of the guinea pig suggests it may also stimulate angiogenesis in this tissue [55]. In the tammar placenta, the bilaminar yolk sac is avascular, while the mesodermal layer of the trilaminar yolk sac differentiates into vascular tissue and mesenchyme. The high relative expression of *IGF2 *in the trilaminar yolk sac suggests that it may be required for growth and vascularisation of the marsupial placenta during the final third of gestation. Although both IGF2 transcript and protein were found in the trilaminar yolk sac, IGF2 antibodies did not react with the mesenchyme or endothelium of vitelline vessels. IGF2 in the mesenchyme may be bound by tissue-specific IGF-BPs that inhibit its interaction with the antibody.

IGF2 may also promote vascularisation of the yolk sac indirectly. Like IGF2, IGF2R was found in the trophoblast and yolk sac endoderm, but not the mesenchyme. These results suggest that the primary targets of IGF2 activity in the yolk sac are the trophoblast and extra-embryonic endoderm. In the eutherian yolk sac, endodermal cells appear critical for the differentiation of mesenchymal cells into angioblasts [56]. Similarly, development of the yolk sac vasculature in the tammar wallaby may require signals from surrounding IGF2-responsive cells (trophoblast and/or yolk sac endoderm).

### IGF2 and VEGF

In the mouse, Vegf is needed for haematopoiesis, differentiation of endothelial lineages, and neo-vascularisation of developing organs including the placenta [45,57,58]. IGF2 may stimulate vascular differentiation of the yolk sac by regulating *VEGF *expression. The present results support this hypothesis. *In vivo *there was a parallel increase in *VEGF *and *IGF2 *expression in the yolk sac during the final third of gestation. Although IGF2 expression declines during days 25 to 26 of gestation while VEGF continues to increase, this is likely to reflect the long half-life of IGF2 protein *in vivo*, where it is maintained in labile pools by IGF2 binding proteins [29,31]. In *vitro, VEGF *expression increased significantly in trilaminar yolk sac explants grown in culture with human-recombinant IGF2. It is possible that IGF2 increased VEGF expression in yolk sac cultures by increasing cellular proliferation, rather than stimulating VEGF expression directly. This study cannot distinguish between these two possibilities. *VEGF *expression in both control and treatment cultures was higher than in the same stages *in vivo*, possibly due to IGF2 contained within the fetal calf serum in the culture medium. The data presented support the suggestion that IGF2 can increase *VEGF *expression either directly or via an increase in cell numbers in the differentiating vascular yolk sac.

### Placental function and IGF2 imprinting

IGF2 is clearly important in the marsupial placenta during late gestation. Moreover, IGF2 is imprinted in the fetus and placenta of the tammar wallaby [14]. Organogenesis and rapid growth occur in the final third of gestation in the tammar, and the metabolic needs of the fetus are greatest at this time [7,8,53,59]. The increase in trilaminar yolk sac area may facilitate efficient transfer of gases and support fetal metabolism during this phase of rapid growth [7-9]. Thus, by stimulating cellular proliferation and survival in the bilaminar and trilaminar yolk sac as well as vascularisation, placental IGF2 may be critical for the fetus to meet its metabolic requirements.

IGF2 may also influence nutrient transport through the yolk sac. In the tammar, glucose transport across the yolk sac increases during the final third of gestation [7,60]. Insulin typically regulates glucose transport, but not in the rodent yolk sac placenta [61,62]. IGF2 may, instead, perform this function in the yolk sac and possibly the chorioallantoic placenta, in which glucose transport is also largely insensitive to insulin [63-66]. In bovine endothelial cells IGF-BP2 enhances glucose transport [67] and IGF2 increases glucose transport in cultured human cytotrophoblasts [68]. However, insulin is also present and imprinted in the marsupial yolk sac [77], so the two may act synergistically.

The placenta is a key site of imprinted gene expression in eutherians and imprinted genes regulate its development and function [69,70]. Presumably the growth promoting functions of IGF2 in the placenta and fetus may explain the maintenance of its imprinting in divergent mammalian species. Further, increased vascular development and growth of the yolk sac is needed to maintain fetal growth and the present study establishes the potential for IGF2 to influence growth and angiogenesis in the placenta of the tammar.

## Conclusion

The expression and proposed functions of IGF2 in the marsupial placenta suggest this gene has a critical role in placentation in all therian mammals. IGF2 appears to increase *VEGF *expression and promote vascularisation of the yolk sac of the tammar. This is the first evidence of a physiological role for an imprinted gene in the placenta of any marsupial. The conserved imprinting of IGF2 in this marsupial with all eutherian species so far investigated, but not in monotremes, suggests that imprinting of this gene may have originated when it acquired a function in the placenta of the therian ancestor.

## Methods

### Animals

Adult female tammars carrying fetuses in the final third of gestation (day 19 to day 26 of a 26.5 day gestation [71]) were euthanised either by cervical dislocation or by an anaesthetic overdose (sodium pentobarbitone, 60 mg/ml, to effect) and portions of the bilaminar (BYS) and trilaminar (TYS) yolk sac collected as previously described [7,53,60]. All experiments were approved by the University of Melbourne Animal Experimentation Ethics Committees and the animal handling and husbandry were in accordance with the CSIRO/Australian Bureau of Agriculture and National Health and Medical Research Council of Australia (1990) guidelines.

### Immunohistochemistry

Immunohistochemistry was performed on matching bilaminar and trilaminar yolk sac samples collected from 13 tammar fetuses in mid to late gestation. Small pieces of endometrium with placenta attached were collected and fixed in 4% PFA before paraffin embedding. Sections (7 μm) were mounted on SuperFrost Plus slides (Menzel-Glaser) before dewaxing and rehydration. A 3 min 0.05% pronase (sigma type XXIV, # *P5147*) antigen retrieval step was required for the IGF2 antibody (Santa Cruz, # *Sc-7435*). IGF1Rα (Santa Cruz, IGF-IRα, #*Sc-712*) and IGF2R (Santa Cruz, # *Sc-14408*) antibodies required a 5 min wash in 0.1% Triton-X-100. Details on the antibodies used are presented in Table 1. Sections were blocked for 25 min at room temperature in 10% normal serum/TBS/1% BSA. IGF2 and IGF2R were used at a concentration of 0.002 g/L and IGF1Rα at 0.0006 g/L. Sections were incubated overnight at 4°C. A biotinylated secondary antibody (DAKO, # *E0432 *or DAKO, # *E0466*) was used with ABComplex/HRP kit (DAKO, # *K0355*) and colour developed with DAB Chromagen tablets (DAKO, # *S3000*). Sections were counterstained in haematoxylin. Immuno-reactivity was evaluated subjectively using the intensity of brown (DAB) colour development, with strong staining of many/most cells given a grade of 5 reducing to no staining (0). Appropriate control, reactions were run in parallel.

**Table 1:** IGF2, IGF2R, and IGF1R antibody specifics. IgG antibody negative controls used to confirm the specificity of the target antibodies are also shown.

**Target**	**Name**	**Supplier**	**Type**	**Immunogen**	**Epitope**
IGF2	IGF-II (F-20)	Santa Cruz (# *sc-7435*)	Goat polyclonal	Human IGF2	Internal
IGF2R	IGF-IIR (H-20)	Santa Cruz (# *sc-14408*)	Goat polyclonal	Human IGF2R	Internal
IGF1R	IGF-IRα (N-20)	Santa Cruz (# *sc-712*)	Rabbit polyclonal	Human IGF1R (α-subunit)	N-terminus

**IgG control**	**Name**	**Supplier**	**IgG antibody controlled for the following target antibodies.**		

Goat IgG	Normal goat IgG	Santa Cruz (# *sc-2028*)	IGF2 and IGF2R		
Rabbit IgG	Normal rabbit IgG	Santa Cruz (# *sc-2027*)	IGF1R		

### Western blotting

Proteins were extracted from tammar uterus and yolk sac placentas in 1 mL of extraction buffer (0.14 M Tris, 6% SDS, 22.4% glycerol). A 25 mg and 50 mg aliquot of extract was mixed with 1/4 reducing Laemmli sample buffer and boiled for 5 min before separation on a 15% SDS-poly-acrylamide gel for 50 mins at 170 V. Protein was transferred to nitrocellulose (in 40% Methanol, Tris-glycine transfer buffer) for 30 min at 50 V followed by 30 min at 100 V at 4°C. Following overnight blocking in 5% skim milk in Tris buffered saline containing 0.05% Tween 20 (SM-TTBS) at 4°C, the membrane was incubated with IGF2 antibody (as used for immunohistochemistry) at a final concentration of 3 mg/mL in SM-TTBS for 1.5 hours at room temperature. The membrane was washed and incubated in HRP conjugated donkey-anti-goat secondary diluted 1:10,000 in SM-TTBS for 45 min at room temperature. The signal was detected with ECL reagent and visualized on Hyperfilm-ECL (GE Healthcare).

### Non-quantitative RT-PCR

Approximately 300 ng of DNase treated (DNA-free, Ambion, # 1906) total RNA (GenElute Mammalian Total RNA Kit, Sigma, # *RTN70*) was used in an Oligo (dT)_12–18 _primed cDNA synthesis reaction (SuperScript First Strand Synthesis System for RT-PCR, Invitrogen, # *11904-018*). Approximately 5 ng of cDNA was used with *IGF2 *primers (Suzuki et al, 2004) (Table 2). PCR was performed with an initial incubation at 94°C for 2 min, 39 cycles of 94°C for 1 min, 60°C for 1 min, and 72°C for 1 min. Primers for *IGF2R *were designed using sequence provided by Professor F. Ishino and Primer3 software (Table 2). *IGF2R *PCR conditions where the same as *IGF2 *PCR but annealing was carried out at 55°C. Promega Taq polymerase B (# *M1661*) and accompanying reagents were used at concentrations of 1.5 mM MgCl_2_, 0.2 mM each dNTPs, and 0.2 μM each primers.

**Table 2:** IGF2 and IGF2R primer sequences for non-quantitative RT-PCR.

**Primer**	**Sequence (5' to 3')**
IGF2 Fw	CCTTTGTGGTGGGGAACTGG
IGF2 Rv	GGATGGGGTCTTCGCTGGGCA
IGF2R Fw	CGAAATAAGACTGCCACTACA
IGF2R Rv	TTAGAGGAAGAGGAAAACAC

### Quantitative RT-PCR

Matched bilaminar and trilaminar yolk sac samples for quantitative PCR were collected from 23 individuals and all were assessed. However, two of these samples were excluded from further analysis due to consistent variations within triplicate samples. mRNA levels were measured for *IGF2, IGF2R*, and *VEGF *(sequence provided by Dr. Laura Parry, The University of Melbourne). cDNA was synthesised as described above. SYBR green (Quantitect, # *204143*) was used in a quantitative PCR on the MJ Research Opticon 2 thermocycler. PCR conditions and primer sequences for target genes are given in Table 3. All primers crossed intron-exon boundaries. *B-ACT *was used as an endogenous gene controland calibrator (forward primer 5' GATCCATTGGAGGGCAAGTCT 3' and reverse primer 5' CCAAGATCCAACTACGAGCTTTTT 3'). Reactions were performed in triplicate and the data analysed in Microsoft Excel and Systat. The amplification efficiency was calculated from the standard curve and Ct values corrected [72-74].

**Table 3:** Quantitative RT-PCR primer sequences and reaction conditions for the target genes *IGF2*, *IGF2R*, and *VEGF*. Melting curve analyses were performed after each PCR and one sample from each triplicate was assessed by gel electrophoresis to confirm there was no contamination.

	***IGF2***	***IGF2R***	***VEGF***
Fw primer 5' to 3'	CCTTTGTGGTGGGGAACTGGT	CACAGGAGGTGGAAATGGTGAA	GATGTCTATCAACGCAGCTACT
Rv primer 5' to 3'	GGATGGGGTCTTCGCTGGGCA	CCCAGAGGCACTGAATAACTT	TGATGTTGTGCACCTCATAGGG

**Protocol**			

	50°C 10 min	50°C 10 min	50°C 10 min
	95°C 15 min	95°C 15 min	95°C 15 min

39 ×	95°C 30 sec	95°C 30 sec	95°C 30 sec
	60°C 20 sec	55°C 20 sec	55°C 20 sec
	72°C 1 min	72°C 40 sec	72°C 40 sec
	86°C 1 sec	75°C 1 sec	76°C 1 sec
	Plate read	Plate read	Plate read

### Tissue culture

Three day 19 and five day 21 fetuses were collected and the yolk sac dissected under sterile conditions at 37°C in either yolk sac fluid or medium (DMEM/Pen-Strep/L-Glutamine/10% FCS). Trilaminar yolk sac portions (2 × 2 mm for day 19 and 4 × 4 mm for day 21) were obtained from each conceptus. Explants were grown in Nunc plates coated with 1.7 % agar in either base medium or base medium containing human recombinant-IGF2 (Chemicon, # *GF007*) in an environment of 6% CO_2 _(BOC gases) and air (20% O_2_: 75% N_2_). Cultures were grown at 37°C in an air-jacketed incubator (Steri-cycle CO_2 _incubator – Hera filter). Based on culture of mouse tissue and the binding efficiency of kangaroo IGF2R for eutherian IGF2, hr-IGF2 was added at a concentration of 100 ng/ml (diluted in sterile filtered PBS/1% BSA) [46,75,76]. IGF2-treated and control explants from day 19 were cultured for 8 hours and then snap-frozen in liquid nitrogen, while day 21 explants were cultured for 18 hours before snap-freezing. Quantitative RT-PCR as described above established relative levels of *VEGF *expression in control and treated yolk sac explants.

### Statistical analyses

Statistical analyses (means, variation, Bonferroni adjusted t-tests) were performed using Microsoft Excel. Repeated measures analyses of variance and multiple comparison tests were conducted using Systat Version 10.2. Quantitative data are presented as means ± s.e.m. unless otherwise indicated. Statistical significance was at the 5% level. An α-value between 0.05 and 0.1 was considered to show a trend worth further consideration.

## Authors' contributions

All authors contributed to the design of the study. MBR, GS, AJP, EIA, and other members of the Renfree Research Group collected the samples; EIA performed all the experiments. All authors read, modified and approved the final manuscript.
